# Evaluation of the Perceived Persuasiveness Questionnaire: User-Centered Card-Sort Study

**DOI:** 10.2196/20404

**Published:** 2020-10-23

**Authors:** Nienke Beerlage-de Jong, Hanneke Kip, Saskia Marion Kelders

**Affiliations:** 1 Department of Psychology, Health and Technology Center for eHealth and Wellbeing Research University of Twente Enschede Netherlands; 2 Department of Medical Microbiology and Infection Prevention University Medical Centre Groningen Groningen Netherlands; 3 Department of Research Stichting Transfore Deventer Netherlands; 4 Optentio Research Focus Area North-West University Vanderbijlpark South Africa

**Keywords:** eHealth, behavior change support systems, card sort, perceived persuasiveness, persuasive systems design, mental model, questionnaire evaluation

## Abstract

**Background:**

eHealth technologies aim to change users’ health-related behavior. *Persuasive* design and system features can make an eHealth technology more motivating, engaging, or supportive to its users. The Persuasive Systems Design (PSD) model incorporates software features that have the possibility to increase the persuasiveness of technologies. However, the effects of specific PSD software features on the effectiveness of an intervention are still largely unknown. The Perceived Persuasiveness Questionnaire (PPQ) was developed to gain insight into the working mechanisms of persuasive technologies. Although the PPQ seems to be a suitable method for measuring subjective persuasiveness, it needs to be further evaluated to determine how suitable it is for measuring perceived persuasiveness among the public.

**Objective:**

This study aims to evaluate the face and construct validity of the PPQ, identify points of improvement, and provide suggestions for further development of the PPQ.

**Methods:**

A web-based closed-ended card-sort study was performed wherein participants grouped existing PPQ items under existing PPQ constructs. Participants were invited via a Massive Open Online Course on eHealth. A total of 398 people (average age 44.15 years, SD 15.17; 251/398, 63.1% women) completed the card sort. Face validity was evaluated by determining the item-level agreement of the original PPQ constructs. Construct validity was evaluated by determining the construct in which each item was placed most often, regardless of the original placement and how often 2 items were (regardless of the constructs) paired together and what interitem correlations were according to a cluster analysis.

**Results:**

Four PPQ constructs obtained relatively high face validity scores: perceived social support, use continuance, perceived credibility, and perceived effort. Item-level agreement on the other constructs was relatively low. Item-level agreement for almost all constructs, except perceived effort and perceived effectiveness, would increase if items would be grouped differently. Finally, a cluster analysis of the PPQ indicated that the strengths of the newly identified 9 clusters varied strongly. Unchanged strong clusters were only found for perceived credibility support, perceived social support, and use continuance. The placement of the other items was much more spread out over the other constructs, suggesting an overlap between them.

**Conclusions:**

The findings of this study provide a solid starting point toward a redesigned PPQ that is a true asset to the field of persuasiveness research. To achieve this, we advocate that the redesigned PPQ should adhere more closely to what persuasiveness is according to the PSD model and to the mental models of potential end users of technology. The revised PPQ should, for example, *enquire if* the user thinks anything is done to provide task support but not *how* this is done exactly.

## Introduction

Owing to the increasing pressure on health care systems, an increasing number of health-related interventions are making use of technology. This is also referred to as eHealth: the use of technology to support health, well-being, and (the organization of) health care [1]. Many eHealth technologies aim to change their users’ health-related attitudes or behaviors. A known pitfall of such technologies is that users stop using them rather quickly or do not use the technology as intended by the developers [2]. This issue of nonadherence can limit the effectiveness of technology because of the dose-response relationship, that is, the more a technology is used, the more effective it is [2]. Furthermore, limited use of technology also hampers sustainability. To deal with this downside of eHealth, it is pivotal that users of eHealth technologies are continuously motivated and engaged by the technology itself. To that end, eHealth technologies can make use of certain design and system features that can make technology more motivating, engaging, and supportive to its users, in turn helping them become *persuasive* eHealth technologies [3-7]. Persuasive (eHealth) technologies may be defined as “computerized software or information systems designed to reinforce, change or shape attitudes or behaviors or both without using coercion or deception” [8]. Despite the potential benefits of persuasion, insight into whether, why, for whom, and how persuasive eHealth technology works remains limited. Most of the existing research has focused on whether persuasive eHealth technology as a whole is effective, which provides little insight into what it is that makes it effective and what the role of persuasiveness is [9]. Thus, there is a need to open the black box of persuasive eHealth technologies to gain insight into whether and how persuasive technology actually increases adherence and whether persuasiveness is related to the effectiveness of such technologies.

### How Does Persuasion Work?

Although we currently have a broad sense of which persuasive strategies are preferred by certain user groups (eg, based on personality traits, age, or gender) [10-14], more research is needed to unravel why and how persuasive technologies work for different types of users. To evaluate the effect of persuasion, we need to gain insight into whether technology users actually experience the included persuasive software features (eg, personalization and social comparison) as persuasive. After all, the persuasive strategies that designers apply in the software may not be perceived as such by the users [15]. It has been stated that the persuasive powers of a technology are determined by an individual’s subjective evaluation of that technology and its impact on the self [16]. This highlights the importance of the subjective experience of persuasiveness as opposed to merely checking whether certain persuasive features are present according to the researchers or developers of the technology. It would therefore be extremely helpful for persuasive technology researchers and developers to have an instrument at hand that enables them to reliably check the persuasive aspects of technologies, not from a designer’ s perspective but from a user’s perspective. This would not only support developers in making their designs more persuasive but also provide the necessary step to evaluate whether technology increases adherence and effectiveness through the persuasiveness of the technology as theorized.

### Toward Designing Persuasive Technologies

For developers and designers, the Persuasive Systems Design (PSD) model was created as a guideline for the development of persuasive eHealth technologies [17]. It provides 4 categories of software features that can be applied to persuasive systems: *primary task support*, *dialogue support*, *social support*, and *credibility support* [17] (Table 1). The model posits that all these features have the ability to increase the persuasiveness of technologies. However, as previously indicated, the effects of specific software features on the persuasiveness of an intervention are still largely unknown. Thus, measuring persuasion would require a measurement tool to evaluate the subjective (*perceived*) persuasion of a technology for the individual user. There are 3 newly developed questionnaires that might play a role in achieving this goal. First, there is the Persuadability Inventory [18]; this is a questionnaire aimed at measuring how susceptible an individual is to certain persuasive strategies such as rewards or social comparison. Although this is an interesting focus area, it does not measure the effects of specific software features on different individuals and, therefore, cannot be used for evaluating the persuasiveness of a system. The second related questionnaire is the Persuasive Potential Questionnaire [19]. This questionnaire is aimed at measuring the potential of different systems to persuade users, especially at the stage when the system is not fully developed. As such, it also does not evaluate the effect of specific software features within a system and seems to focus on how persuadable an individual is. Therefore, this questionnaire seems ill suited for the goal of evaluating the persuasiveness of a system. The third questionnaire is the Perceived Persuasiveness Questionnaire (PPQ) [16,20]. The PPQ is a 31-item scale that assesses perceived persuasiveness according to the 4 categories of the PSD (primary task support, dialogue support, social support, and credibility support) and the 4 related constructs: unobtrusiveness, effort, effectiveness, and (overall) perceived persuasiveness [16,20].

Although the goal of the PPQ is not clearly articulated in the first papers that it appeared in, it has been used to evaluate how users perceive the persuasiveness of a certain system (a digital weight loss intervention) and how the different included constructs relate to each other within a structural equation model. Later studies have used the PPQ to evaluate the persuasive effects of different virtual agents [21] in a web-based weight loss intervention [22] and in an antibiotic information app for nurses [23]. Thus, the questionnaire has been applied to evaluate interventions as a whole or specific persuasive elements within such interventions, and it has been applied to the general public and health care professionals as well. Overall, as the intent of the PPQ is to measure perceived persuasiveness, it is interesting to evaluate to what extent the scale can be used to assess the perceived persuasiveness of technologies.

Therefore, this study addresses questions related to how to evaluate and measure the dimensions of persuasion in eHealth. The PPQ is recognized as a promising low-threshold instrument to measure perceived persuasiveness among a heterogeneous set of people [16,20]. However, to live up to its potential, it is important to evaluate whether the PPQ truly matches the underlying PSD model and the mental models of its broad range of potential target groups [15,24]. A mental model is a person’s simplified representation of how something works in the real world. As the PPQ focuses on how persuasiveness is *perceived,* it is even more essential that its items actually match its users’ mental models. Consequently, this study aims to evaluate the face and construct validity of the PPQ constructs to identify points of improvement for a potentially updated version. To achieve this, a broad target group with an interest in eHealth was asked to evaluate the face and construct validity of the PPQ constructs by means of a card-sort study. This provides insight into the extent to which the PPQ items are grouped under the PPQ constructs as intended by its developers and as such provide face validity. Furthermore, we explore the extent to which participants see the constructs included in the PPQ as separate from each other and the items as fitting with each other, which provides information on construct validity. Together, this provides insight into whether the PPQ items are capable of rendering a valid impression of a technology’s perceived persuasiveness and whether the PPQ items match the mental models of the users of the technology. The outcomes of this study can be used to establish whether any changes are needed to the PPQ constructs and items.

**Table 1:** Overview of the categories of the Persuasive Systems Design model.

Category	Description	Examples of design features
Primary task support	What the technology does to support the user in carrying out his primary task	Reduction, tunneling, tailoring, personalization, self-monitoring, simulation, rehearsal
Dialogue support	How the technology supports its users via computer-human communication	Praise, rewards, reminders, suggestions, similarity, liking, social role
Social support	How the technology uses social influence to motivate its users	Social facilitation, social comparison, normative influence, social learning, cooperation, competition, recognition
Credibility support	How the technology’s design contributes to instilling trust in its users	Trustworthiness, expertise, surface, credibility, real-world feel, third-party endorsements, verifiability

## Methods

### Design

Traditionally, the card-sorting methodology is used to create and evaluate a fit between an information structure and the mental models of its target group such that it enables them to easily find, interpret, and apply information [25-27]. However, previous research has indicated that it can also yield valuable insights into how mental models fit with other kinds of structuring of information, such as the items of a questionnaire [28].

Specifically, in this study, we applied the card-sort methodology to evaluate the face and construct validity of a questionnaire. The questionnaire’s face validity refers to the extent to which its items subjectively (at first glance) seem to actually cover the constructs they are intended to measure [29]. In the card sort, this was ascertained by evaluating how many participants had grouped each individual item in the intended construct. The questionnaire’s construct validity represents the extent to which the included items are actually related to each other and the construct they intend to measure [30]. In the card sort, this was evaluated by analyzing how the items are grouped and placed in constructs by the respondents themselves, according to their own mental models regardless of the constructs that the items originally belonged to. This cross-sectional study used a closed-ended card sort to evaluate the face and construct validity of the PPQ. In this type of card-sort study, the categories that have to be used to group items have already been defined beforehand. During the card-sort study, participants were asked to sort cards with, in this case, questionnaire items into the PPQ constructs (primary task support, dialogue support, perceived credibility, perceived social support, perceived unobtrusiveness, perceived persuasiveness, perceived effort, perceived effectiveness, and use continuance) in a way that was logical or meaningful to them. The resulting groups render information about the participants’ mental models concerning these constructs, including an agreement or disagreement among users [31].

### Participants

Participants were invited to join the study via a Massive Open Online Course (MOOC) on *eHealth: Combining Psychology, Technology and Health* that was hosted by the research group of the authors of this paper [32]. Anyone with an interest in eHealth technology can join the MOOC, free of charge. It is targeted at an international audience via the FutureLearn platform [33]. Participants of the MOOC were invited to participate in the fourth week (of a total of 6 weeks) of the MOOC. This week of the course was entirely focused on the persuasive design of eHealth technology. In addition, at that point of the web-based course, participants had already finished reading and practicing with information about eHealth, human-centered design, and behavior change. This means that they had acquired at least some basic knowledge about eHealth and persuasive design. After completing the lessons of week 4, participants were presented with a short introductory text including disclosure about the research (Multimedia Appendix 1). They were invited to participate on a voluntary basis in this study. Data were collected during 10 runs of the MOOC, which ran from May 23, 2016, to December 8, 2019. A total of 12,439 people actively participated in the MOOC during this time span. A total of 602 people started filling in the questionnaire, of which 398 (251/398, 63.1% women; 147/398, 36.1% men) completed the study and were included. The average response rate per MOOC run was 3.35% (398/11,880) of the active *learners* of the MOOC. The participants’ mean age was 44.15 years (SD 15.17) with participants’ age ranging from 18 to 81 years. Most participants (288/398, 72.4%) were *somewhat familiar* with eHealth. However, despite having joined the MOOC, some (17/398, 4.3%) participants indicated that they were not familiar with eHealth at all. The first participant completed the card sort on June 11, 2016, and the last participant completed the card sort on June 25, 2019. Most (121/398, 30.4%) of the participants had joined the MOOC during its very first run. Participants’ data were included in the data analysis if all demographic questions were answered and the card sort was completed.

### Materials and Procedure

**Figure 1:**
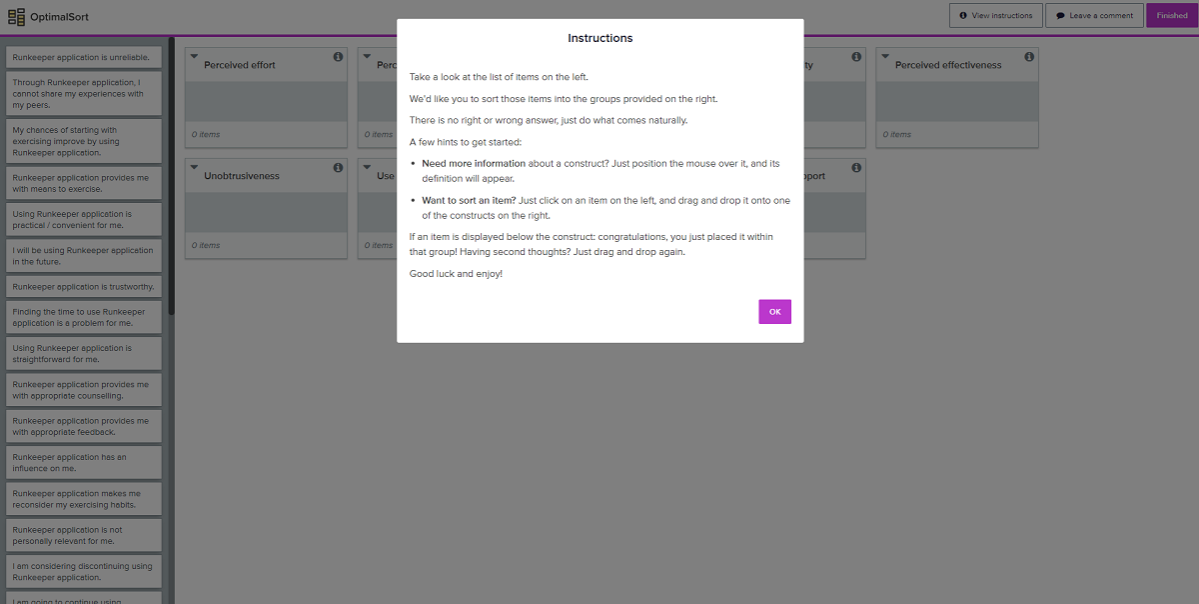
Screenshot of card sort software during instructions.

**Figure 2:**
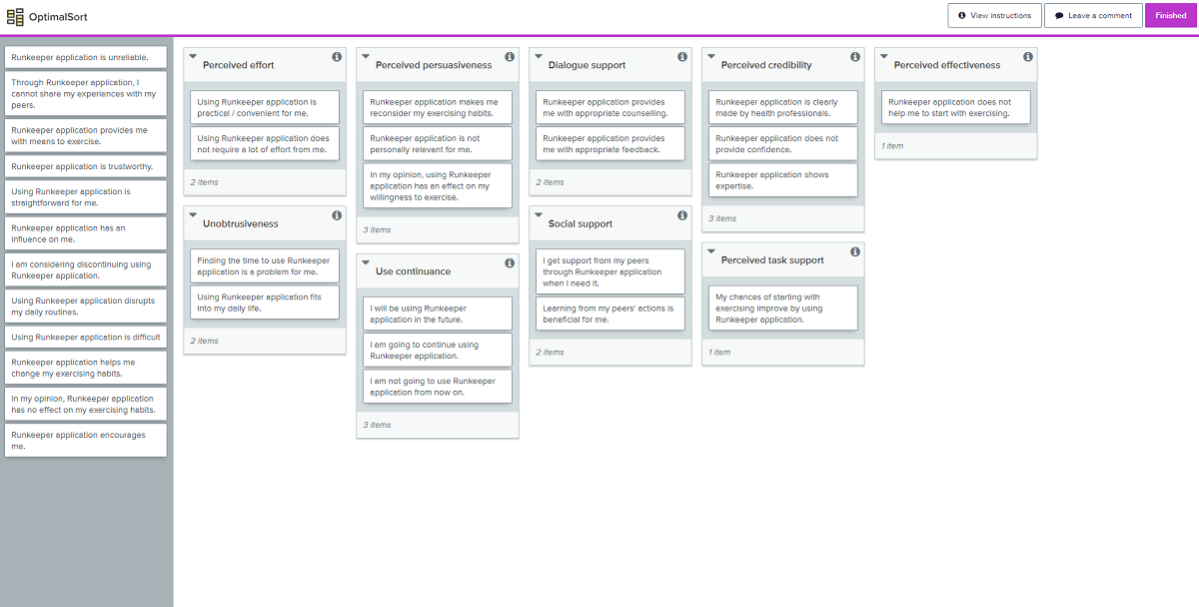
Screenshot of OptimalSort software during card sorting task.

All participants received an explanation of the background, purpose, and methodology of the study. Optimal workshop software [34] was used for this study. This software allowed us to inquire about the participants’ demographics (ie, gender, age, and familiarity with eHealth) and complete the card sort at a single (web) location. The software provided participants with instructions on how to perform the card sort and how to sort the cards by a simple drag-and-drop functionality. Screenshots of the card-sort software used are provided in Multimedia Appendix 2. Figure 1 portrays the instructions page; Figure 2 shows a screenshot during the actual sorting task.

The focal point of this study’s card sort was the PPQ. Overall, the PPQ consists of 31 items divided over 9 constructs. The constructs, their meaning, and an example item are provided in Table 2. A complete overview of the PPQ constructs and their items is provided in Multimedia Appendix 3. To make the items easier to interpret and less abstract, for this study, the PPQ items were formulated around an existing, well-known eHealth technology: the Runkeeper app [35]. However, the items can be adapted to fit other persuasive technologies. Participants did not need to actively work with the Runkeeper app; it was merely used as a way to concretize the PPQ constructs. For example, it allowed a card to state “Runkeeper application does not help me to start with exercising” rather than “XYZ does not help me change [target behavior].”

During the card sort, the participants were asked to *drag and drop* all PPQ items onto the PPQ constructs to group them into a meaningful collection of items. They were assured that there were no right or wrong answers and to *just do what comes naturally*. It was explained to them that they could see the definition of each PPQ construct by hovering the mouse over it. During the card sort, the software ensured that participants had to select a single construct to place an item in. Completing the card-sort task took the participants an average of 10.55 min (SD 8.03).

**Table 2:** Short overview of Perceived Persuasiveness Questionnaire constructs and examples of their items.

PPQ^a^ construct (abbreviation)	Short description	No. of items	Example of an item
Primary task support (TASK)	Whether the technology helps to achieve the goal	3	Runkeeper helps me change my exercising habits
Perceived dialogue support (DIAL)	Whether the technology provides feedback and guidance to the user	3	Runkeeper provides me with appropriate counselling
Perceived credibility (CRED)	The perceived reliability and trustworthiness of the technology	5	Runkeeper is clearly made by health professionals
Perceived social support (SOCI)	Whether the technology allows the user to share with and learn from their peers	3	I get support from my peers through Runkeeper when I need it
Perceived persuasiveness (PERS)	Whether users think that the technology is valuable and has an influence on them	3	Runkeeper has an influence on me
Perceived unobtrusiveness (UNOB)	How disturbing the technology is to daily life	4	Using Runkeeper disrupts my daily routines
Perceived effort (EFFO)	The endeavor that the technology entails	3	Using Runkeeper is difficult
Perceived effectiveness (EFFE)	The efficacy of the technology	3	My chances of starting with exercising improve by using Runkeeper
Use continuance (CONT)	Willingness of users to adopt the technology in the future	4	I will be using Runkeeper in the future

^a^PPQ: Perceived Persuasiveness Questionnaire.

### Data Analysis

All card-sort data were exported into an Excel spreadsheet (Microsoft) that was specifically developed for the analysis of card sorts [36]. This spreadsheet was extended, adapted, and used in this study and previous research from our research group [24,26,37].

Face validity was evaluated by determining the item-level agreement of the original PPQ constructs. This was done by evaluating how many participants had grouped each individual item in the intended construct by means of descriptive statistics. As a second step for the data analysis, the focus was on construct validity of the PPQ constructs. It is of interest to see how the items are grouped and placed in constructs by the respondents themselves, according to their own mental models, regardless of the constructs that the items originally belonged to.

Third, we performed a more in-depth analysis by exploring how often any 2 items (regardless of the constructs) were paired together and by measuring interitem correlations (a measure of how consistently the items of that construct are grouped together). To study the items’ coherence regardless of the clusters, we performed a cluster analysis of the items. These analyses provide insight into which items were perceived as measuring a similar construct. We performed a hierarchical cluster analysis (intervals based on squared Euclidian distance) using IBM SPSS 24.

## Results

### Face Validity—Item-Level Agreement in the Original PPQ

The first research question focused on the face validity of the PPQ constructs, revolving around the question of the extent to which the participants’ sorts fit the original PPQ constructs. Item-level agreement on these constructs is shown in Table 3.

Some of the original PPQ constructs demonstrated a relatively strong (average >50%) item-level agreement, indicating that most of the participants grouped the *correct* items within these constructs. Agreement about the items of the constructs perceived social support, use continuance, perceived credibility, and perceived effort was high. On the other hand, item-level agreements for the constructs perceived task support, perceived dialogue support, perceived usefulness, perceived persuasiveness, and perceived effectiveness were relatively low. Agreement on these items was more diffused with respondents frequently mingling with these constructs. Some interesting observations from Table 3 are as follows:

- The items of the perceived task support construct were grouped relatively often into the constructs of perceived persuasiveness and perceived effectiveness.
- The items of perceived dialogue support were grouped relatively often under perceived task support.
- The items of perceived unobtrusiveness were also grouped under perceived effort.
- The items of perceived persuasiveness were also grouped relatively often under perceived effectiveness and perceived task support.
- The items of perceived effectiveness were most frequently also grouped under the construct perceived persuasiveness and perceived task support.

**Table 3:** Item-level agreement in original Perceived Persuasiveness Questionnaire placements.

Original construct	Item	TASK^a^ (%)	DIAL^b^ (%)	CRED^c^ (%)	SOCI^d^ (%)	UNOB^e^ (%)	PERS^f^ (%)	EFFO^g^ (%)	EFFE^h^ (%)	CONT^i^ (%)
TASK	24	56	4	2	0	2	11	5	16	4
TASK	15	25	4	3	1	6	15	7	36	4
TASK	23	20	3	2	1	1	22	4	43	4
DIAL	26	22	63	2	3	0	4	1	5	1
DIAL	21	22	54	5	9	1	3	1	5	1
DIAL	11	17	29	1	2	0	33	5	8	5
CRED	29	1	1	90	0	1	4	1	2	1
CRED	6	2	2	61	0	7	4	5	14	6
CRED	4	3	2	88	1	1	3	1	2	1
CRED	16	11	7	46	1	6	12	3	12	3
CRED	17	3	1	89	1	1	3	0	2	0
SOCI	22	3	6	1	88	1	1	0	1	1
SOCI	20	3	8	1	78	4	1	2	1	3
SOCI	30	2	4	1	88	1	1	0	2	1
UNOB	10	5	2	1	1	69	3	8	5	8
UNOB	14	4	1	1	1	64	2	17	4	7
UNOB	1	5	2	2	1	36	6	25	16	8
UNOB	28	3	1	1	1	35	2	39	3	15
PERS	18	5	4	1	1	2	53	3	28	3
PERS	5	10	3	5	1	11	23	6	29	12
PERS	19	17	7	3	0	1	42	3	24	3
EFFO	2	4	2	1	0	15	2	71	3	3
EFFO	7	6	3	3	1	18	5	49	9	6
EFFO	31	7	3	1	1	11	4	65	5	5
EFFE	3	18	2	3	0	2	26	6	38	6
EFFE	12	11	1	2	1	3	42	5	32	4
EFFE	25	8	2	4	0	4	9	5	63	4
CONT	27	1	1	2	0	1	5	3	5	83
CONT	13	2	1	3	0	1	6	2	8	76
CONT	8	2	1	1	1	5	3	4	8	76
CONT	9	2	1	2	0	5	4	6	7	74

^a^TASK: primary task support.

^b^DIAL: perceived dialogue support.

^c^CRED: perceived credibility.

^d^SOCI: perceived social support.

^e^UNOB: perceived unobtrusiveness.

^f^PERS: perceived persuasiveness.

^g^EFFO: perceived effort.

^h^EFFE: perceived effectiveness.

^i^CONT: use continuance.

### Optimal Item-Level Agreement

As a second step, focusing on construct validity, we calculated in what constructs the items were most often placed regardless of the original placements of the items. An overview of the average agreement within the constructs for the original constructs versus the constructs as they should be according to popular placements is given in Table 4.

**Table 4:** Overview of the agreement within the original constructs versus popular placement constructs.

Construct	Average agreement within the original PPQ^a^ constructs	Average agreement within constructs as defined by popular placement
Perceived task support	33.7	73.7
Perceived dialogue support	48.7	73.7
Perceived credibility	74.8	74.8
Perceived social support	84.7	84.7
Unobtrusiveness	51.0	56.3
Perceived persuasiveness	39.3	42.5
Perceived effort	61.7	56.0
Perceived effectiveness	44.3	41.8
Use continuance	77.3	77.3

^a^PPQ: Perceived Persuasiveness Questionnaire.

According to popular placement (Table 5), the items of the constructs perceived task support and perceived dialogue support should be merged into a single category with a high average agreement (>70%). This merged category showed much higher agreement than the items within the separate constructs according to the original PPQ. Existing constructs with high agreement according to popular placement were perceived credibility support, perceived social support, and use continuance. All these constructs had the same average agreement as when using the original placement, with their items remaining unchanged. Agreement with popular placement for unobtrusiveness and perceived persuasiveness was somewhat higher than agreement within these constructs of the original PPQ. Simultaneously, agreement with popular placement for perceived effort and perceived effectiveness was slightly lower than agreement within the original PPQ. For all these constructs and for the merged construct, the composition of items would have to change based on popular placements.

**Table 5:** Popular placement matrix.

Item	TASK^a^ (%)	DIAL^b^ (%)	CRED^c^ (%)	SOCI^d^ (%)	UNOB^e^ (%)	PERS^f^ (%)	EFFO^g^ (%)	EFFE^h^ (%)	CONT^i^ (%)
24	56	4	2	0	2	11	5	16	4
26	22	63	2	3	0	4	1	5	1
21	22	54	5	9	1	3	1	5	1
29	1	1	90	0	1	4	1	2	1
17	3	1	89	1	1	3	0	2	0
4	3	2	88	1	1	3	1	2	1
6	2	2	61	0	7	4	5	14	6
16	11	7	46	1	6	12	3	12	3
22	3	6	1	88	1	1	0	1	1
30	2	4	1	88	1	1	0	2	1
20	3	8	1	78	4	1	2	1	3
10	5	2	1	1	69	3	8	5	8
14	4	1	1	1	64	2	17	4	7
1	5	2	2	1	36	6	25	16	8
18	5	4	1	1	2	53	3	28	3
12	11	1	2	1	3	42	5	32	4
19	17	7	3	0	1	42	3	24	3
11	17	29	1	2	0	33	5	8	5
2	4	2	1	0	15	2	71	3	3
31	7	3	1	1	11	4	65	5	5
7	6	3	3	1	18	5	49	9	6
28	3	1	1	1	35	2	39	3	15
25	8	2	4	0	4	9	5	63	4
23	20	3	2	1	1	22	4	43	4
3	18	2	3	0	2	26	6	38	6
15	25	4	3	1	6	15	7	36	4
5	10	3	5	1	11	23	6	29	12
27	1	1	2	0	1	5	3	5	83
8	2	1	1	1	5	3	4	8	76
13	2	1	3	0	1	6	2	8	76
9	2	1	2	0	5	4	6	7	74

^a^TASK: primary task support.

^b^DIAL: perceived dialogue support.

^c^CRED: perceived credibility.

^d^SOCI: perceived social support.

^e^UNOB: perceived unobtrusiveness.

^f^PERS: perceived persuasiveness.

^g^EFFO: perceived effort.

^h^EFFE: perceived effectiveness.

^i^CONT: use continuance.

### Cluster Analysis of the Items

The results described in the sections *Face Validity—Item-Level Agreement in the Original PPQ* and *Optimal Item-Level Agreement* have indicated that some changes in the PPQ would be advisable based on the mental models of the participants. In the following section, we therefore performed an additional cluster analysis of the items to study their coherence regardless of the constructs. Multimedia Appendix 4 shows an item-item matrix of correlations. Within the table, we indicate which construct most of the items in each cluster belong to. There are 3 relatively strong clusters that are identical to the original perceived credibility support, perceived social support, and use continuance. However, the placement of the other items was much more spread out over the other clusters (Multimedia Appendix 4). Some interesting observations are as follows:

- The original perceived effort and perceived unobtrusiveness could still be found unchanged in the item-item matrix. However, the items of perceived effort also showed overlap with 2 other items (items 28 and 1) that were mainly grouped in the latter. Merging the 2 constructs would lead to an interitem correlation of 0.898, which is slightly lower than perceived effort and higher than perceived unobtrusiveness.
- On the basis of cluster analysis, the original construct of perceived dialogue support could be reduced to only 2 items. This change slightly improved the interitem correlation from 0.715 to 0.731.
- The items of the original constructs perceived task support, perceived effectiveness, and perceived persuasiveness showed rather strong overlap and were grouped into a single cluster.
- One item of perceived dialogue support (item 11) had been moved to a
  left over
  cluster alongside item 24, which was originally part of perceived task support.

## Discussion

### Principal Findings

This study set out to evaluate the PPQ by means of a card sort of its items to determine whether the current version of the PPQ can be used to assess the overall perceived persuasiveness of a technology according to the mental model of its users. The results show that some constructs within the PPQ seem to have high face and construct validity in their current form (ie, perceived credibility, social support, and use continuance), whereas other constructs (ie, perceived task support, perceived dialogue support, perceived effort, unobtrusiveness, effectiveness, and persuasiveness) have less face validity and overlap with other PPQ constructs. It appears that the PPQ in its current form does not fit the mental models of the participants, which sheds doubt on the usefulness of the scale to assess perceived persuasiveness because of the subjective nature of this concept. The results of this study provide a first step toward a thorough redesign of the PPQ to more robustly and validly measure constructs related to perceived persuasiveness in a manner that fits the perceptions and experiences of its potential users.

First, it is noticeable that the constructs perceived task support and perceived dialogue support show strong overlap. Both constructs are part of the PSD model but differ in their theoretical underpinning, meaning, and effect [16,17]. However, it seems as if the items are not able to distinguish between the constructs. The PPQ should be able to make a distinction between these constructs as their theoretical aims differ from one another: perceived task support covers the features of the technology that aim to support its user in achieving its goals, whereas perceived dialogue support covers computer-human interactions that are enabled by the technology [16,17]. In line with this, it has been shown that the effect of including elements of perceived task support and perceived dialogue support in technologies is different; for example, perceived dialogue support has been shown to be related to adherence, although this relationship was not found for perceived task support. Thus, the items within these constructs need to be redefined to enable adequate measurement of these distinct constructs. When looking at the items, it is apparent that the conceptual level of the items in the different constructs varies. For perceived task support, the items are aimed at the overall goal of the concept (eg, “Runkeeper helps me change my exercising habits”), whereas for perceived dialogue support, the items are framed on separate elements within the concept of perceived dialogue support (eg, “Runkeeper provides me with appropriate counseling”). It might be that the focus on separate elements in perceived dialogue support does not sufficiently address the overall goal of the category, in this case supporting the dialogue between the system and the user. Therefore, we suggest redefining all items of the PPQ such that they represent a similar level of abstraction, focusing on the purpose of the construct rather than on how it might have been applied in a technology.

Second, the constructs perceived effort and perceived unobtrusiveness also show strong overlap. Ideally, the PPQ should include all constructs that are part of a technology’s persuasiveness. However, it should omit constructs that seem to predict rather than being a part of persuasiveness. Perceived effort revolves around how strenuous it is to use the technology, regardless of its context. In addition, perceived effort is seen to be part of usability, which is a precondition for persuasion but not persuasion itself [16]. Therefore, we argue that perceived effort should not be a part of the PPQ. Perceived unobtrusiveness, on the other hand, concerns the extent to which the technology can be used as a *seamless part of daily routines* [16]. From many previous studies, we know that the success of a technology not only depends on the technology itself but also on its fit with the use context [38,39]. A main way of technology to be persuasive is, therefore, to increase the fit of the technology in daily life and by improving the way in which it is integrated into the work or thought process of its users. Therefore, we advocate that perceived unobtrusiveness should remain part of the PPQ and that *unobtrusiveness support* might even be seen as a fifth category of the PSD model.

Third, perceived effectiveness, perceived persuasiveness, and (to a lesser extent) perceived task support display strong overlap. This might be explained by the fact that they all cover the technology’s support toward achieving one’ s goals. However, it is noticeable that of these constructs, only perceived task support is part of the PSD model. Furthermore, the construct of perceived task support is focused on how supportive the technology is, whereas perceived effectiveness and perceived persuasion are concerned with the extent to which technology-driven support has actually contributed to a behavior change. As the latter two are more of an outcome measure of persuasion than a form of persuasion itself, we advocate that they should not be part of the PPQ. For perceived persuasiveness, this is also confirmed in the literature, where perceived persuasiveness was shown to be the result of perceived task support, perceived dialogue support, perceived credibility, and perceived unobtrusiveness. Yet, perceived persuasiveness itself did not influence any variables except the (intended) use of the technology, suggesting that it is an outcome rather than a part of persuasion [16]. This confirms our belief that perceived persuasiveness should merely be seen as an outcome of persuasion, rather than being part of it. Moreover, it seems peculiar to have a construct called perceived persuasiveness as a part of a questionnaire with the same name. This suggests that the other constructs are not part of the same theoretical concept and should thus not be included in the questionnaire.

Fourth, the construct use continuance has and maintains strong face and construct validity. However, following the same reasoning as described above, it is questionable whether this construct is part of perceived persuasion or is more of an outcome or effect of perceiving persuasion. Moreover, we argue that use continuance would be a measure of adherence rather than persuasion and therefore advocate that it should not be part of the PPQ.

### Using a Card-Sort Study to Evaluate the Questionnaire

In this study, we opted to use the card-sort method to investigate the face and construct validity of the PPQ instead of more traditional methods such as interviews and focus groups. A large advantage of the card-sort method is that the data of more people can be combined in a robust and statistical manner, more so than using qualitative methods [40]. In this manner, we could statistically analyze whether participants agreed with the original grouping of the PPQ items and which items were clustered together in new user-centered concepts. A disadvantage of the card-sort method is that the reasoning behind participants’ clustering is not taken into account, whereas in a qualitative study, a researcher is able to focus on this more. Therefore, a conscious decision on which method to use in a particular context should be made.

Although the card sort yielded many valuable results in this study, it would also have been useful to use this closed-ended card sort at an earlier stage: during the development of the PPQ, before its release. In this manner, a card sort can be used to verify whether assumptions made by the researchers on the structure of the questionnaire and clustering of the items are in line with the users’ mental models. Consequently, card sorting is not merely a suitable method to evaluate existing questionnaires but can also be used as a development tool when creating new questionnaires.

Furthermore, in this study, prospective end users of the PPQ were invited to participate in the card sort study. An advantage of this approach is that the resulting structure of a questionnaire fits the mental models of users, who might lack the necessary expertise to make decisions based on theoretical underpinning of the constructs. Consequently, we recommend combining user-based card sorts with expert-based card sorts to combine both perspectives, resulting in a theory-based and user-centered questionnaire.

Finally, in this study, we used a closed-ended card sort, which means that the *final* categories were presented to the users. For a new and improved version of the PPQ, in a later stage of development, an open-ended card sort might be used, in which participants are asked to cluster items together individually and provide them with a label of their own choice to cross-reference the construct validity.

Overall, card sort is a very promising method for questionnaire construction and evaluation. However, more research that applies this method and reflects on its benefits and barriers is required to study its full potential at every stage of questionnaire development, redesign, and evaluation.

### Future Research

Further steps must be taken to redesign and validate the PPQ. First, an expert evaluation of the concept of perceived persuasiveness should be carried out to assess what subconstructs need to be included in this broad concept and which items can be used to assess these subconstructs. Second, measures such as Cronbach alpha and interitem correlations are relevant for its reliability, and a factor analysis is needed to evaluate its (convergent and divergent) construct validity. Furthermore, the predictive validity of the PPQ for adherence or effectiveness should be investigated.

Following such research, it would be highly relevant to also explore the relatedness of perceived persuasiveness with other factors, such as engagement [41], adherence [42] and the effectiveness of technologies. This would allow future research to address the following relevant questions: To what extent does perceived persuasiveness matter? Does it actually make its users more adherent? Does it actually improve the effects that are achieved through using the technology? Can the PPQ be used at early stages during the developmental stages of eHealth technologies to make them as persuasive as possible?

### Strengths and Limitations

A strength of this study is that the PPQ is assessed from the user perspective and insight is yielded in the mental models of users for this valuable concept. However, the card sort method did not allow us to assess the theoretical content validity of the scale and the subconstructs. The results showed that this is a step that still needs to be taken. However, this study did allow us to explore whether the PPQ holds enough potential value to invest more resources in a more extensive validation process.

The results of this study should be interpreted with caution. First, the participants of this study were no experts in the field of persuasion. Thus, although definitions of the constructs were provided, it is not certain whether they had enough skills to truly grasp the meaning of the PPQ constructs and items. However, the choice to include these nonexperts as participants was a conscious one. After all, the PPQ is intended to be filled in by technology users just like them, not by experts in the field of persuasion.

Simultaneously, because of the highly international nature of the participants, it is not known if language barriers might have influenced the results. However, given the fact that the participants were recruited via an English language MOOC, it can be assumed that their English language skills are at least sufficient to understand the meaning of written text.

Finally, it is noticeable that the participants were not as consistent in their groupings as they could have been. For example, items 25 and 12 are reversed versions of the same item but are not most frequently grouped together (by only 131/398, 32.9% of the respondents). However, this could also be a symptom of the overlap that participants perceived between different constructs.

### Conclusions

In summary, we believe that the original PPQ in combination with the findings of this study, provide a solid starting point toward a redesigned PPQ that is a true asset to the field of persuasiveness research. It has the potential to contribute to answering the all-important question of *what works when for whom*. However, to be able to achieve this, we advocate that the redesigned PPQ should adhere more closely to what persuasiveness is and to the mental models of potential end users of technology.

In its current form, the PPQ covers many broad constructs, with items that are very specific and diverse. We suggest altering the items to be less focused on details and specific design features and more focused on what the technology is intended to do per category of the PSD model. In other words, the PPQ should enquire *if* the user thinks anything is done to provide task support, but not *how* this is done exactly. This would also increase the reliability and external validity of PPQ measurements. After all, considering the breadth of technologies and the endless varieties of possible target groups, aims, and contexts of these technologies, it is nearly impossible to cover all possible ways of providing (for example) task support. Therefore, there is a need for a new, more abstract version of the PPQ that closely matches the concept of perceived persuasiveness and fits with the mental models of its users.

## Appendix

Multimedia Appendix 1 Card sort invitation from the Massive Open Online Course .

Multimedia Appendix 2 Screenshots of the card sort procedure.

Multimedia Appendix 3 Original Perceived Persuasiveness Questionnaire constructs and associated cards.

Multimedia Appendix 4 Item×Item groupings according to cluster analysis (in percentage of respondents).

## Abbreviations

MOOC: Massive Open Online Course
PPQ: Perceived Persuasiveness Questionnaire
PSD: persuasive systems design
