# Supplementary material for: Evaluation of the Perceived Persuasiveness Questionnaire: User-Centered Card-Sort Study
Source: J Med Internet Res. 2020 Oct 23;22(10):e20404. doi: 10.2196/20404 (PMC7647815; doi:10.2196/20404)
Supplement: Multimedia Appendix 1 [file jmir_v22i10e20404_app1.docx]

### Appendix A

| How persuasive do *you* think it is?  We’ll end this week on behavior change and persuasive technology with a request to you, our learners. Are you willing to participate in one of our research projects? The project focuses on the development of an evaluation tool for the perceived persuasiveness of eHealth technologies.  Low adherence remains a well-known challenge for the success of eHealth technologies all over the world.  As you know, persuasion can be used to improve adherence and the success of eHealth interventions.  Yet, it often does not achieve this goal and the effect of particular persuasive elements on eHealth success is to large extent still unknown.  Thus far, little is known about what does determine whether persuasive elements work or do not work. On the contrary: many questions remain unanswered. For example:   - Do people notice the persuasive elements of an intervention? - And even more so: is that awareness even necessary for the persuasiveness to have an effect?   To be able to answer such questions, Oinas-Kukkonen et al. have developed the PPQ: the Perceived Persuasiveness Questionnaire. It is based on the PSD (Persuasive Systems Design) model and measures whether eHealth users notice persuasive elements in an eHealth intervention.  Currently, the questionnaire is being evaluated in a cooperation between the University of Oulu (Finland) and ourselves (the Center of eHealth and Wellbeing Research of the University of Twente, the Netherlands).  As part of this evaluation, we are now taking the first steps towards studying whether the items of the questionnaire are suitable to measure the constructs they are supposed to represent. Already, this has shown us very interesting results but we are interested in further enlarging our study population.  **And that is where you come in!**  Are you willing and able to spend a bit of your time (about 10 minutes) to help us progress our research? We would be very grateful if you would participate in an online card sort, to evaluate the PPQ.  We hope you will participate! Thank you in advance and in anyway: good luck with and we hope you enjoy the rest of the online course!  **This link directs to a Card Sort study by Nienke Beerlage-de Jong, PhD of University of Twente, the Netherlands. The outcomes of the study are used for research purposes and for the further development of the PPQ. The outcomes may be published, but anonymity of all data is guaranteed. No prior knowledge is needed to participate, and the very simple procedure will be explained to you. Of course, we are more than happy to keep you informed about the outcomes of our study. If you are interested, please state your e-mail address on the final screen of the card sort study. Your e-mail address will not be used for any other purpose.** |
| --- |
