# Supplementary material for: Evaluation of the Perceived Persuasiveness Questionnaire: User-Centered Card-Sort Study
Source: J Med Internet Res. 2020 Oct 23;22(10):e20404. doi: 10.2196/20404 (PMC7647815; doi:10.2196/20404)
Supplement: Multimedia Appendix 2 [file jmir_v22i10e20404_app2.docx]

### Appendix B

Screenshots of the Card Sort software (1) during instruction and (2) during the sorting task.


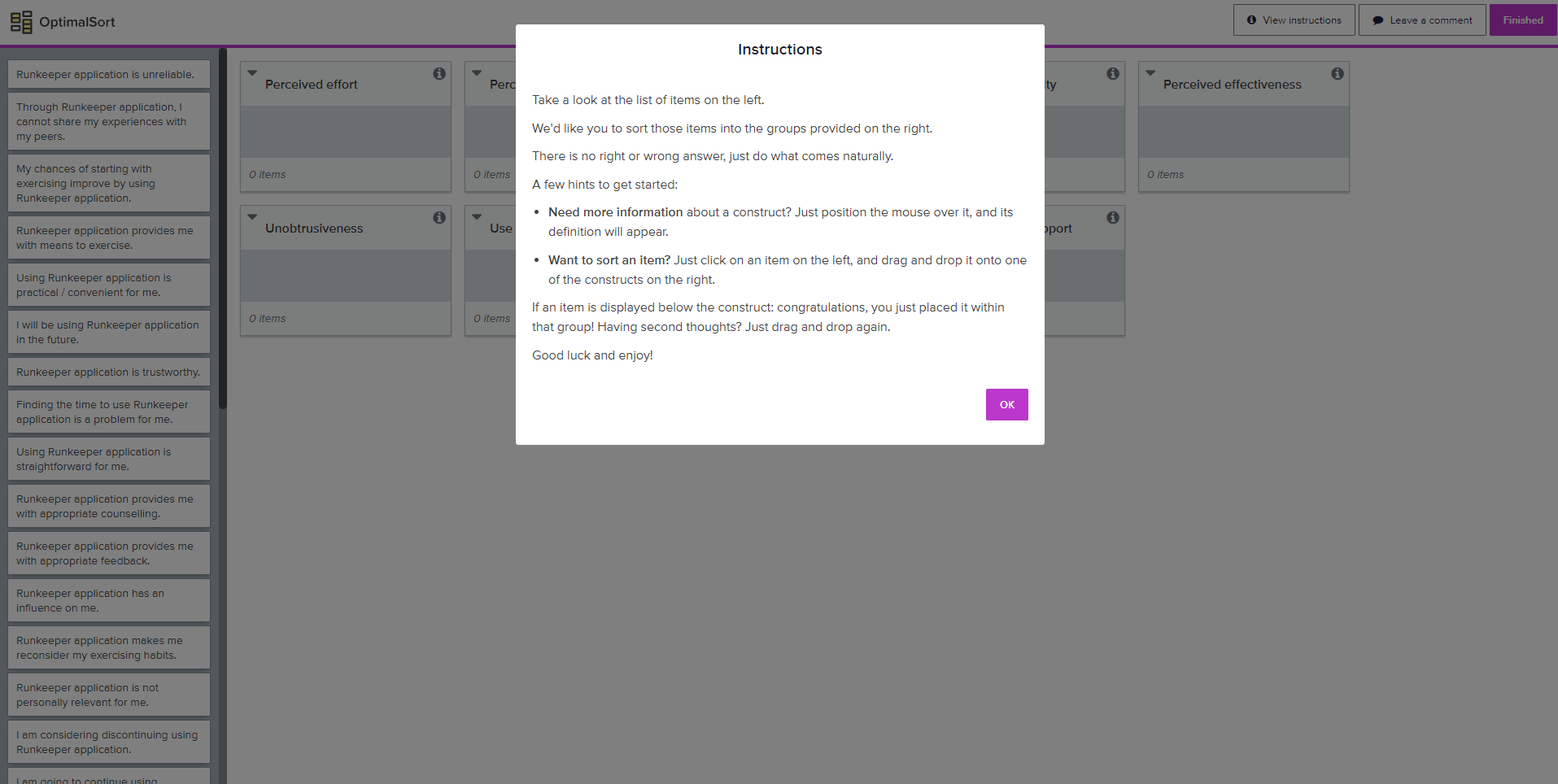


Figure 1. Screenshot of card sort software during instructions


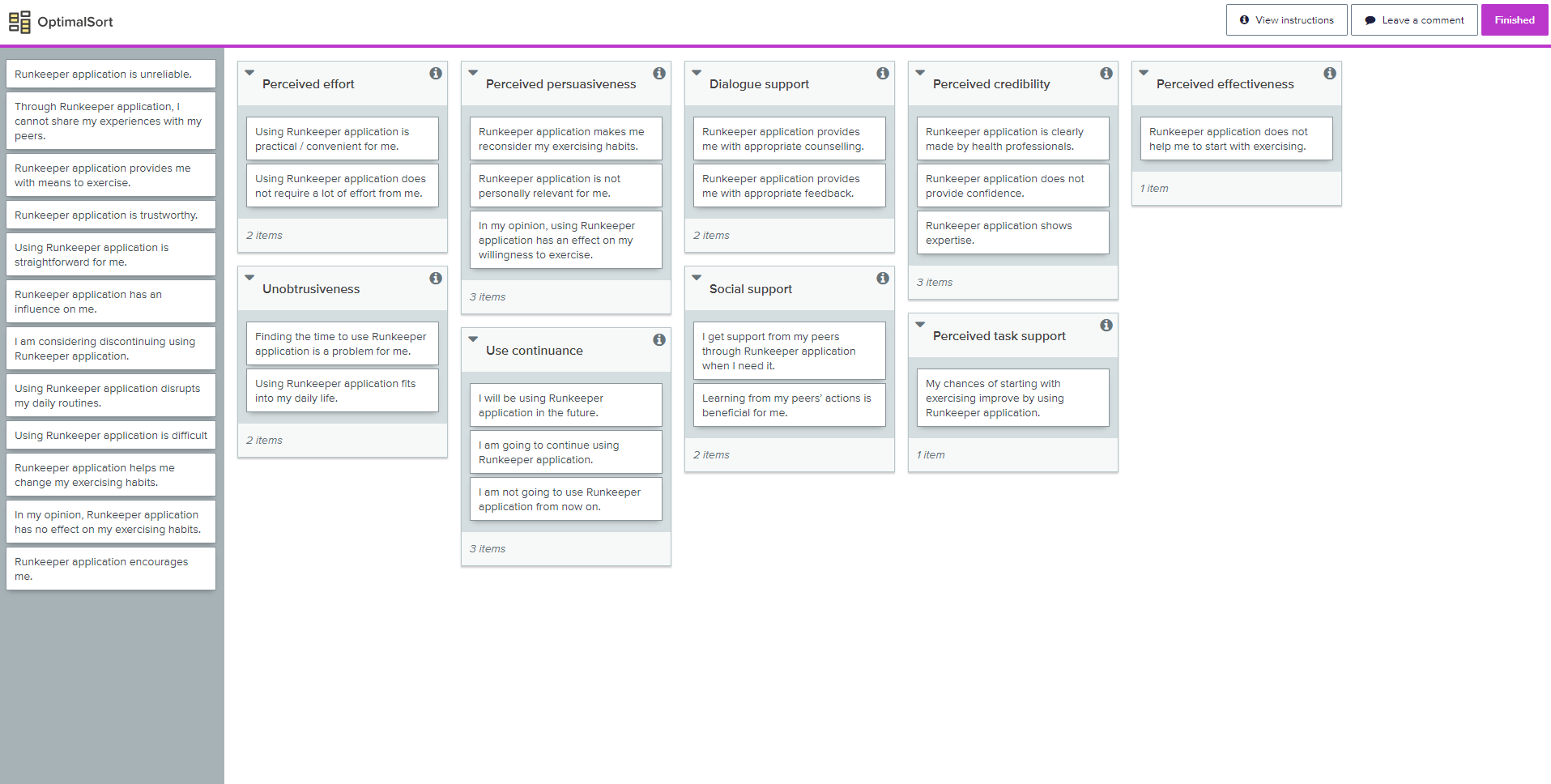


Figure 2. Screenshot of OptimalSort software during card sorting task.
