# Supplementary material for: Evaluation of the Perceived Persuasiveness Questionnaire: User-Centered Card-Sort Study
Source: J Med Internet Res. 2020 Oct 23;22(10):e20404. doi: 10.2196/20404 (PMC7647815; doi:10.2196/20404)
Supplement: Multimedia Appendix 3 [file jmir_v22i10e20404_app3.docx]

### Appendix C

Original PPQ constructs and associated cards

| **Construct** | **Card no** | **Original item** |
| --- | --- | --- |
| Primary task support | 24 | 1. XYZ^1^ provides me with means to [lose weight]^2^. |
|  | 15 | 2. XYZ helps me [lose weight]. |
|  | 23 | 3. XYZ helps me change [my eating habits]. |
| Dialogue support | 26 | 1. XYZ provides me with appropriate feedback. |
|  | 21 | 2. XYZ provides me with appropriate counseling. |
|  | 11 | 3. XYZ encourages me. |
| Perceived credibility | 29 | 1. XYZ is trustworthy. |
|  | -6 | 2. XYZ is reliable. |
|  | 4 | 3. XYZ shows expertise. |
|  | -16 | 4. XYZ instills confidence. |
|  | 17 | 5. XYZ is clearly made by health professionals.^3^ |
| Social support | 22 | 1. I get support from my peers through XYZ when I need it. |
|  | -20 | 2. Through XYZ, I can share my experiences with my peers. |
|  | 30 | 3. Learning from my peers’ actions is beneficial for me. |
| Unobtrusiveness | 10 | 1. Using XYZ fits into my daily life. |
|  | 14 | 2. Using XYZ disrupts my daily routines. (Reversed item) |
|  | 1 | 3. Using XYZ, is practical / convenient for me. |
|  | -28 | 4. Finding the time to use XYZ is not a problem for me. |
| Perceived persuasiveness | 18 | 1. XYZ has an influence on me. |
|  | -5 | 2. XYZ is personally relevant for me. |
|  | 19 | 3. XYZ makes me reconsider [my eating habits]. |
| Perceived effort | 2 | 1. Using XYZ does not require a lot of effort from me. |
|  | 7 | 2. Using XYZ is straightforward for me. |
|  | 31 | 3. Using XYZ is laborious. (Reversed item) |
| Perceived effectiveness | 3 | 1. My chances of [losing weight] improve by using XYZ. |
|  | 12 | 2. In my opinion, using XYZ has an effect on [my weight]. |
|  | 25 | 3. In my opinion, XYZ has no effect on [my weight]. (Reversed item) |
| Use continuance | 27 | 1. I am going to continue using XYZ. |
|  | 13 | 2. I will be using XYZ in the future. |
|  | 8 | 3. I am considering discontinuing using XYZ. (Reversed item) |
|  | 9 | 4. I am not going to use XYZ from now on. (Reversed item) |

^1^ The name of the system should go here.

^2^ The intent should go here.

^3^ This item has been changed from “XYZ is made by health professionals”.
