# Supplementary material for: Evaluation of the Perceived Persuasiveness Questionnaire: User-Centered Card-Sort Study
Source: J Med Internet Res. 2020 Oct 23;22(10):e20404. doi: 10.2196/20404 (PMC7647815; doi:10.2196/20404)
Supplement: Multimedia Appendix 4 [file jmir_v22i10e20404_app4.docx]

Table 6. Item x Item groupings according to cluster analysis (in % of respondents)

| **Item** | **17** | **29** | **4** | **6** | **16** | **22** | **30** | **20** | **8** | **9** | **13** | **27** | **2** | **31** | **7** | **10** | **14** | **28** | **1** | **21** | **26** | **12** | **18** | **19** | **3** | **23** | **15** | **25** | **5** | **11** | **24** |
| --- | --- | --- | --- | --- | --- | --- | --- | --- | --- | --- | --- | --- | --- | --- | --- | --- | --- | --- | --- | --- | --- | --- | --- | --- | --- | --- | --- | --- | --- | --- | --- |
| 17 | 100 | 84 | 84 | 58 | 43 | 2 | 2 | 1 | 1 | 2 | 3 | 2 | 2 | 2 | 3 | 2 | 1 | 2 | 1 | 5 | 2 | 1 | 2 | 3 | 3 | 3 | 3 | 4 | 4 | 1 | 2 |
| 29 | 84 | 100 | 84 | 60 | 44 | 1 | 2 | 1 | 2 | 3 | 4 | 3 | 1 | 1 | 4 | 2 | 1 | 2 | 3 | 5 | 2 | 4 | 4 | 5 | 5 | 4 | 4 | 4 | 4 | 2 | 3 |
| 4 | 84 | 84 | 100 | 57 | 42 | 1 | 2 | 1  **CRED**^a^ | 2 | 2 | 3 | 2 | 1 | 2 | 3 | 1 | 1 | 1 | 1 | 5 | 4 | 3 | 3 | 3 | 4 | 4 | 4 | 4 | 5 | 4 | 3 |
| 6 | 58 | 60 | 57 | 100 | 42 | 1 | 1 | 6 | 11 | 13 | 6 | 6 | 3 | 9 | 7 | 3 | 10 | 10 | 5 | 4 | 4 | 5 | 4 | 7 | 7 | 8 | 14 | 18 | 16 | 3 | 4 |
| 16 | 43 | 44 | 42 | 42 | 100 | 1 | 3 | 5 | 9 | 9 | 5 | 4 | 3 | 7 | 5 | 2 | 8 | 7 | 4 | 11 | 10 | 10 | 12 | 13 | 9 | 11 | 19 | 17 | 18 | 9 | 10 |
| 22 | 2 | 1 | 1 | 1 | 1 | 100 | 82 | 73 | 1 | 1 | 1 | 1 | 0 | 0 | 1 | 1 | 1 | 1 | 2 | 11 | 5 | 2 | 1 | 2 | 1 | 2 | 0 | 1 | 2 | 4 | 2 |
| 30 | 2 | 2 | 2 | 1 | 3 | 82 | 100 | 73 | 1 | 1 | 1  **SOCI**^b^ | 1 | 1 | 0 | 1 | 2 | 1 | 2 | 2 | 10 | 6 | 2 | 2 | 3 | 2 | 1 | 2 | 1 | 1 | 4 | 1 |
| 20 | 1 | 1 | 1 | 6 | 5 | 73 | 73 | 100 | 6 | 6 | 2 | 2 | 1 | 6 | 3 | 1 | 5 | 7 | 2 | 8 | 6 | 2 | 1 | 1 | 1 | 1 | 6 | 6 | 7 | 2 | 1 |
| 8 | 1 | 2 | 2 | 11 | 9 | 1 | 1 | 6 | 100 | 74 | 64 | 70 | 3 | 9 | 5 | 6 | 11 | 18 | 8 | 2 | 2 | 7 | 3 | 5 | 8 | 5 | 12 | 16 | 19 | 6 | 4 |
| 9 | 2 | 3 | 2 | 13 | 9 | 1 | 1 | 6 | 74 | 100 | 66 | 72 | 4 | 11 | 6 | 6 | 12 | 17 | 7 | 2 | 2 | 7 | 4 | 4 | 6 | 7 | 11 | 12 | 18 | 5 | 4 |
| 13 | 3 | 4 | 3 | 6 | 5 | 1 | 1 | 2 | 64 | 66 | 100 | 72 | 3 | 5 | 6  **CONT**^c^ | 7 | 5 | 11 | 7 | 2 | 2 | 8 | 6 | 5 | 9 | 10 | 7 | 8 | 15 | 6 | 6 |
| 27 | 2 | 3 | 2 | 6 | 4 | 1 | 1 | 2 | 70 | 72 | 72 | 100 | 4 | 5 | 6 | 9 | 7 | 13 | 7 | 2 | 2 | 9 | 5 | 6 | 7 | 7 | 5 | 6 | 14 | 7 | 6 |
| 2 | 2 | 1 | 1 | 3 | 3 | 0 | 1 | 1 | 3 | 4 | 3 | 4 | 100 | 58 | 51 | 20 | 20 | 35 | 32 | 2 | 3 | 5 | 2 | 4 | 8 | 5 | 5 | 5 | 5 | 3 | 7 |
| 31 | 2 | 1 | 2 | 9 | 7 | 0 | 0 | 6 | 9 | 11 | 5 | 5 | 58 | 100 | 48 | 13 | 23 | 36 | 26 | 3  **UNOB + EFFO**^d^ | 4 | 4 | 4 | 3 | 5 | 3 | 11 | 10 | 12 | 5 | 6 |
| 7 | 3 | 4 | 3 | 7 | 5 | 1 | 1 | 3 | 5 | 6 | 6 | 6 | 51 | 48 | 100 | 20 | 17 | 25 | 32 | 4 | 4 | 8 | 6 | 6 | 9 | 9 | 7 | 6 | 8 | 6 | 8 |
| 10 | 2 | 2 | 1 | 3 | 2 | 1 | 2 | 1 | 6 | 6 | 7 | 9 | 20 | 13 | 20 | 100 | 58 | 36 | 41 | 2 | 3 | 6 | 5 | 6 | 7 | 6 | 5 | 5 | 9 | 5 | 6 |
| 14 | 1 | 1 | 1 | 10 | 8 | 1 | 1 | 5 | 11 | 12 | 5 | 7 | 20 | 23 | 17 | 58 | 100 | 48 | 36 | 2 | 3 | 5 | 3 | 4 | 3 | 5 | 12 | 9 | 15 | 3 | 4 |
| 28 | 2 | 2 | 1 | 10 | 7 | 1 | 2 | 7 | 18 | 17 | 11 | 13 | 35 | 36 | 25 | 36 | 48 | 100 | 30 | 2 | 1 | 5 | 2 | 3 | 4 | 4 | 9 | 9 | 13 | 4 | 5 |
| 1 | 1 | 3 | 1 | 5 | 4 | 2 | 2 | 2 | 8 | 7 | 7 | 7 | 32 | 26 | 32 | 41 | 36 | 30 | 100 | 4 | 5 | 8 | 8  **DIAL**^e^ | 9 | 9 | 11 | 7 | 9 | 11 | 6 | 9 |
| 21 | 5 | 5 | 5 | 4 | 11 | 11 | 10 | 8 | 2 | 2 | 2 | 2 | 2 | 3 | 4 | 2 | 2 | 2 | 4 | 100 | 47 | 6 | 5 | 7 | 6 | 6 | 9 | 6 | 5 | 20 | 19 |
| 26 | 2 | 2 | 4 | 4 | 10 | 5 | 6 | 6 | 2 | 2 | 2 | 2 | 3 | 4 | 4 | 3 | 3 | 1 | 5 | 47 | 100 | 5 | 7 | 10 | 5 | 9 | 7 | 5 | 6 | 25 | 14 |
| 12 | 1 | 4 | 3 | 5 | 10 | 2 | 2 | 2 | 7 | 7 | 8 | 9 | 5 | 4 | 8 | 6 | 5 | 5 | 8 | 6 | 5 | 100 | 41 | 36 | 34 | 31 | 29 | 33 | 23 | 24 | 16 |
| 18 | 2 | 4 | 3 | 4 | 12 | 1 | 2 | 1 | 3 | 4 | 6 | 5 | 2 | 4 | 6 | 5 | 3  **TASK + PERS + EFFE**^f^ | 2 | 8 | 5 | 7 | 41 | 100 | 37 | 31 | 32 | 20 | 26 | 23 | 28 | 13 |
| 19 | 3 | 5 | 3 | 7 | 13 | 2 | 3 | 1 | 5 | 4 | 5 | 6 | 4 | 3 | 6 | 6 | 4 | 3 | 9 | 7 | 10 | 36 | 37 | 100 | 29 | 33 | 21 | 21 | 19 | 23 | 25 |
| 3 | 3 | 5 | 4 | 7 | 9 | 1 | 2 | 1 | 8 | 6 | 9 | 7 | 8 | 5 | 9 | 7 | 3 | 4 | 9 | 6 | 5 | 34 | 31 | 29 | 100 | 35 | 28 | 32 | 18 | 19 | 19 |
| 23 | 3 | 4 | 4 | 8 | 11 | 2 | 1 | 1 | 5 | 7 | 10 | 7 | 5 | 3 | 9 | 6 | 5 | 4 | 11 | 6 | 9 | 31 | 32 | 33 | 35 | 100 | 32 | 38 | 19 | 20 | 27 |
| 15 | 3 | 4 | 4 | 14 | 19 | 0 | 2 | 6 | 12 | 11 | 7 | 5 | 5 | 11 | 7 | 5 | 12 | 9 | 7 | 9 | 7 | 29 | 20 | 21 | 28 | 32 | 100 | 41 | 26 | 14 | 24 |
| 25 | 4 | 4 | 4 | 18 | 17 | 1 | 1 | 6 | 16 | 12 | 8 | 6 | 5 | 10 | 6 | 5 | 9 | 9 | 9 | 6 | 5 | 33 | 26 | 21 | 32 | 38 | 41 | 100 | 33 | 9 | 13 |
| 5 | 4 | 4 | 5 | 16 | 18 | 2 | 1 | 7 | 19 | 18 | 15 | 14 | 5 | 12 | 8 | 9 | 15 | 13 | 11 | 5 | 6 | 23 | 23 | 19 | 18 | 19 | 26 | 33 | 100 | 14 | 14 |
| 11 | 1 | 2 | 4 | 3 | 9 | 4 | 4 | 2 | 6 | 5 | 6 | 7 | 3 | 5 | 6 | 5 | 3 | 4 | 6 | 20 | 25 | 24 | 28 | 23 | 19 | 20 | 14  **Other**^g^ | 9 | 14 | 100 | 18 |
| 24 | 2 | 3 | 3 | 4 | 10 | 2 | 1 | 1 | 4 | 4 | 6 | 6 | 7 | 6 | 8 | 6 | 4 | 5 | 9 | 19 | 14 | 16 | 13 | 25 | 19 | 27 | 24 | 13 | 14 | 18 | 100 |

^a^CRED: perceived credibility.

^b^SOCI: perceived social support.

^c^CONT: use continuance.

^d^UNOB: perceived unobtrusiveness; EFFO: perceived effort.

^e^DIAL: perceived dialogue support.

^f^TASK: primary task support; PERS: perceived persuasiveness; EFFE: perceived effectiveness.

^g^Other.
